# Les tuberculomes intracraniens: à propos de 125 cas

**Published:** 2012-07-02

**Authors:** Faycal Moufid, Noureddine Oulali, Nizare El Fatemi, Rachid Gana, Rachid Maaqili, Fouad Bellakhdar

**Affiliations:** 1 Service de Neurochirurgie, CHR Al Farrabi, Faculté de Médecine Oujda, Maroc; 2 Service de Neurochirurgie, Hôpital Ibn Sina Rabat, Maroc

**Keywords:** Tuberculome intracrânien, biopsie stéréotaxique, prise en charge, Maroc

## Abstract

Les tuberculomes intracrâniens représentent l'une des localisations les plus graves de la tuberculose, leur incidence varie en fonction du contexte représentant 0,2% des processus intracrâniens dans les pays occidentaux et 5 à 10% des masses intracrâniennes dans les pays en voie de développement. Nous rapportons une étude rétrospective de 125 cas. L'hypertension intracrânienne (45%) et le déficit neurologique (36%) sont les signes cliniques les plus fréquents. La lésion était localisée dans 60% des cas en sus-tentoriel et dans 40% des cas en sous-tentoriel. L'approche thérapeutique a consisté en un abord direct du tuberculome dans 67 cas (53%), une biopsie stéréotaxique dans 32 cas (25%), le traitement médical en première intention sans confirmation histologique dans 26 cas (20%). Avant 1993 notre service ne disposait pas de cadre de stéréotaxie, notre attitude thérapeutique consistait soit en un abord direct de la lésion dans 70% des cas, soit un traitement antituberculeux en première intention sans confirmation histologique (30%). Cette attitude était corrélée à une mortalité et morbidité non négligeables respectivement 3% et 10%. Après 1993; le taux d'abords direct a chuté a 38%, avec 47% de biopsies stéréotaxiques et seulement 13% des patients traités par antibacillaires sans preuve histologique. Ceci s'est accompagné d'une réduction significative de mortalité a 1,4% (p = 0,0003) et de morbidité a 2% (p = 0,0027).

## Introduction

Les tuberculomes intracrâniens représentent l'une des localisations les plus graves de la tuberculose [1]. Le diagnostique est basé sur un faisceau d'arguments anamnestiques, clinico-biologiques, et radiologiques. La confirmation reste histologique. La prise en charge des tuberculomes intracérébraux a bénéficié des progrès en matière de neuroimagerie avec le développement de la stéréotaxie ainsi que l'efficacité de protocoles actuels de chimiothérapie antibacillaire [2]. Le but de notre travail est d'une part; exposer les aspects épidémiologiques et cliniques ainsi que le protocole de prise en charge de cette entité dans notre contexte; et d'autre part évaluer l'apport de la stéréotaxie dans la prise en charge et le pronostic de cette affection.

## Méthodes

Il s'agit d'une étude rétrospective menée au sein du service de neurochirurgie de l'hôpital Ibn Sina Rabat. L'analyse a concernée 125 patients traités pour tuberculome intracrânien. Ont été exclus de l’étude les dossiers incomplets et les cas d'histologie non concluante. Afin d’évaluer l'impacte de l'introduction en 1993 de la stéréotaxie dans notre protocole de prise en charge; Les malades de notre série ont été divisé en deux groupes; le premier groupe (n = 58) est constitué des malades traités avant 1993, le deuxième les malades traités après cette date (n = 67). Les résultats concernant le taux de confirmation histologique, la durée d'hospitalisation, la morbi-mortalité et les séquelles ont été comparés en les soumettant aux tests statistiques (Khi deux et Student test); la différence est jugée statistiquement significative pour un p

## Résultats

L’âge moyen est de 26 ans (extrêmes 13–65 ans), 56% de nos patients étaient dans leurs 3^ème^ ou 4^ème^ décennie. On a noté une légère prédominance Féminine (69 Femmes et 56 Hommes). Soixante dix pour cent de nos malades étaient de bas niveau socio-économique.

Un contage tuberculeux récent est retrouvé dans 26 cas (20%). Un antécédent tuberculeux confirmé est retrouvé chez trois patients dont 2 traités pour tuberculoses pulmonaire et un pour méningite tuberculeuse. Dans notre série un seul patient était immunodéprimé (splénectomisé).

Le délai diagnostic moyen est de 6 mois (3 semaines à 4 ans). Les signes généraux (fièvre et altération de l’état général) ont été observés chez 21 patients (17%). Un syndrome d'hypertension intracrânienne a été observé dans 56 cas (45%), un déficit moteur dans 46 cas (36%), un syndrome cérébelleux dans 22 cas, et une épilepsie dans 27 cas. Une atteinte ophtalmologique a été observée chez 34 patients sous forme d'une baisse de l'acuité visuelle dans 15 cas, et une cécité dans 19 cas. Enfin 3 patients ont été admis avec des troubles de conscience. Une tuberculose extracérébrale concomitante a été observé dans 24 cas (20%); ceci sous forme d'une tuberculose pulmonaire dans 15 cas, tuberculose vertébrale dans 7 cas et 2 cas de tuberculose ganglionnaire.

Les examens biologiques ont montré une vitesse de sédimentation (VS) accélérée dans 80%; une Leucopénie dans 8% des cas; une intradermoréaction a la tuberculine positive chez 28% des patients. Aucun de nos patients n'avait une sérologie HIV positive. Tous nos malades ont bénéficié d'une TDM cérébrale avec injection de produit de contraste; par contre 43 cas seulement (34%) ont été explorés par une IRM. La lésion était localisée en sus-tentoriel dans 73 cas (60%) dont 8 étaient multiples. Chez 52 (40%) malades le tuberculome avait un siège sous tentoriel dont 11 au niveau du tronc cérébral. Deux localisations rares ont été notées (Le sinus caverneux et l'angle ponto-cérébélleux). Dans 113 cas (90%) de notre série le tuberculome était unique; alors que les tuberculomes multiples ont été retrouvés chez 12 patients (10%). Les tuberculomes ont été divisés en trois groupes en fonction de leurs diamètre; 83 (66%) avaient un diamètre compris entre 2 et 3 cm, 36 tuberculomes (28%) mesuraient plus de 3 cm et dans 6 cas la lésion mesurait moins de 2 centimètres.

**Figure 1:**
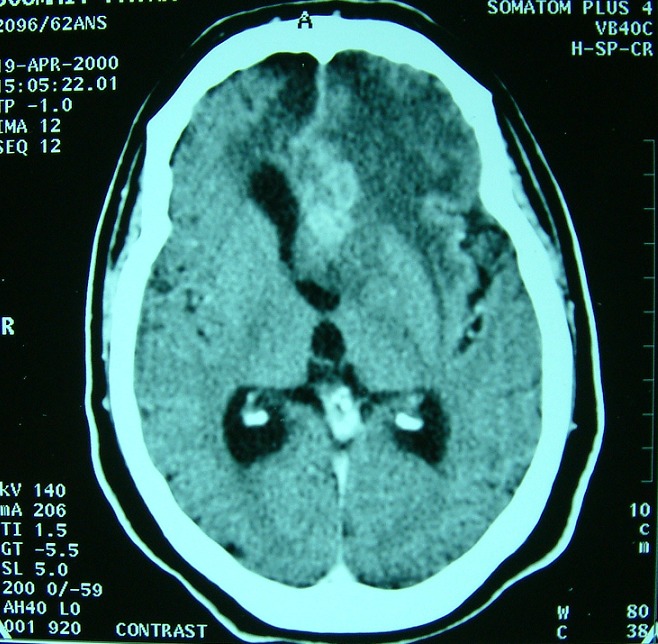
TDM cérébrale après injection de produit de contraste montrant un processus frontal médian d'aspect hétérogène au sein d'une hypodensité frontale, ce processus exerce un effet de masse sur la ligne médiane, et se rehausse de façon intense

**Figure 2:**
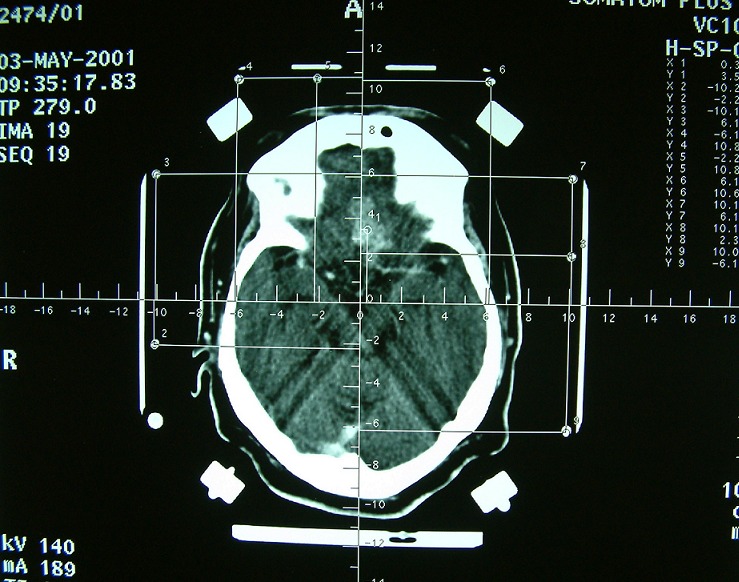
Biopsie en condition stéréotaxique du processus, ayant confirmé le diagnostic de tuberculome

**Figure 3:**
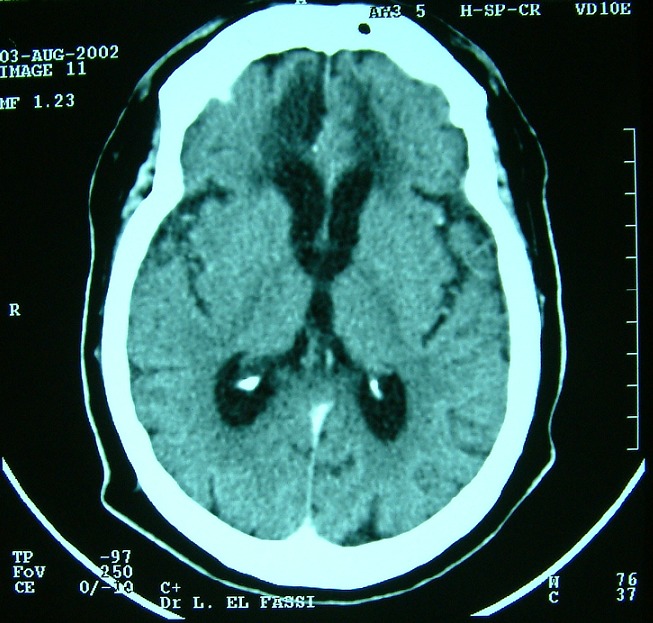
TDM cérébrale après injection du produit de contraste, après 1 an de traitement antibacillaire montrant la régression du tuberculome, avec persistance d'une zone d'hypodensité frontale

Les lésions revêtent différents aspects radiologiques. L'aspect typique évocateur de tuberculome n'est retrouvé que dans 43 cas (34%). Alors que dans la majorité des cas le tuberculome prend un aspect non spécifique pouvant évoquer une pathologie tumorale ou inflammatoire (Figure 1, Figure 2, Figure 3). Une hydrocéphalie associée a été retrouvée chez 14 patients (11%), tous avaient des lésions infra-tentorielles. Dans notre série l'approche thérapeutique a consisté en: Un abord direct du tuberculome dans 67 cas (53%) avec: Exérèse complète dans 42 cas, Incomplète dans 35 cas ; une biopsie stéréotaxique dans 32 cas (25%) ; le traitement médical en première intention sans confirmation histologique dans 26 cas (20%) ; la chirurgie de l'hydrocéphalie dans 14 cas avec 12 dérivations ventriculo-péritonéale et 2 drainages ventriculaires externes.

Avant 1993 on ne disposait pas de cadre de stéréotaxie dans notre service ce qui explique que le choix thérapeutique ne peut se faire qu'entre l'abord direct (70% des cas) et le traitement antibacillaire sans confirmation histologique (30%). Après introduction de la stéréotaxie dans notre protocole de prise en charge en 1993; le taux d'abords direct a chuté a 38%, avec 47% de biopsies stéréotaxiques et seulement 13% des patients traités par antibacillaire sans preuve histologique (Tableau 1). Le taux global de confirmation histologique est passé de 70% à 83% après 1993 (p = 0,005). Tous nos patients ont reçu le même protocole de chimiothérapie antibacillaire. Avant 1993 nous avons enregistré un taux de morbidité péri-opératoire de 10% et un taux de mortalité de 3%. Après 1993 on a constaté une réduction statistiquement significative des taux de morbidité et de mortalité respectivement 2% (p: 0,0003) et 1,4% (p: 0,0027) (Tableau 2). Le recul moyen est de 9 ans (2 ans et 20 ans). L’évolution au long terme est marquée par la persistance de séquelles neurologiques sous forme de déficit moteur d'importance variable et de comitialité chez 21 malades. Dont 11 patients (19%) du groupe traités avant 1993 et 10 patients (15%) du 2^ème^ groupe, cependant cette différence reste non significative (p = 0,2525).

**Tableau 1:** L’évolution de notre stratégie thérapeutique avant et après la survenue de la stéréotaxie

	Avant 1993	Après 1993
Abord chirurgical direct	41 (70%)	26 (39%)
Biopsie stéréotaxique	0	32 (48%)
Traitement antibacillaire sans preuve histologique	17 (30%)	9 (13%)

**Tableau 2:** Comparaison des résultats du protocole de prise en charge avant et après introduction de la stéréotaxie en 1993, objectivant l'amélioration statistiquement significative du taux de confirmation histologique, de mortalité et de morbidité post opératoire

	Avant 1993	Après 1993	P
Confirmation histologique (taux global)	41 cas (70%)	58 cas (83%)	0,0005
Durée moyenne d'hospitalisation	23 jours	14 jours	0,0028
Mortalité péri-opératoire	3 (3%)	1 (1,4%)	0,0027
Morbidité post chirurgicale	6 (10%)	2 (2%)	0,0003
Séquelles	11 cas (19%)	10 cas (15%)	0,2525

## Discussion

La tuberculose constitue un problème de santé publique majeur dans les pays en voie de développement et notamment au Maroc ou l'on constate une incidence annuelle de 30000 nouveau cas / an toutes localisations confondues.

Un tuberculome est une masse de tissu granulomateux tuberculeux ayant été contenue et limitée par les défenses immunitaires de l'hôte. Il se présente comme une lésion expansive, intracrânienne [3, 4]. Son incidence varie en fonction de la zone géographique, rare dans les pays occidentaux ou son incidence varie de 0,5- 2% [5] des processus intracrâniens; par contre il est assez fréquent dans les pays du tiers monde ou il représente entre 5 à 10% des masses intracrâniennes [6, 7]. Cependant avec la pandémie du SIDA on assiste a une émergence de cet entité dans les grands centres urbains des pays développés ou la haute densité de population et la sélection de mycobactéries résistantes contribuent a l'augmentation de l'incidence des tuberculomes intracrâniens.

Le tuberculome intracrânien résulte d'une diffusion hématogène à partir d'un foyer primitif généralement pulmonaire. Sur le plan étiopathogénique on note l'absence de bacilles acido-alcoolo-résistants (BAAR) dans le tuberculome, les mycobactéries n'ont pour rôle que de déclencher la réaction immunitaire à médiation cellulaire. Plusieurs tubercules se constituent puis fusionnent pour former une lésion souvent lobulée [6]. L'examen histologique du tuberculome montre une nécrose caséeuse centrale entourée de cellules épithéliales géantes de Langhans, lymphocytes et de polynucléaires. Cette étiopathogénie explique la constitution à distance de la primo-infection tuberculeuse et l'absence de Bacille de Koch (BK) dans les prélèvements [7]. La symptomatologie est non spécifique dépendant de la localisation, la taille et le nombre des lésions. Les signes généraux (fièvre ou fébricule) dans les semaines précédents les signes neurologiques sont inconstants. La recherche d'une autre localisation tuberculeuse doit être systématique; la fréquence d'une localisation évolutive concomitante varie de 25 à 40% dans la littérature [8]. Dans notre série on a retrouvé un antécédent de tuberculose chez 3 malades et une tuberculose extra cérébrale évolutive chez 24 patients soit 20% des cas, dominés par la tuberculose pulmonaire (15 cas) et les spondylodiscites (7cas). Cependant l'absence d'une tuberculose extracérébrale ne doit ne aucun cas écarter le diagnostic de tuberculome. Au scanner aucune image n'est spécifique du tuberculome, bien que l'aspect le plus typique est une lésion hypodense avec prise de contraste périphérique en couronne, associé parfois à des calcifications centrales réalisant la classique image en cible. Cet aspect n'est ni constant (retrouvé seulement chez 34% de nos malades) ni spécifique évoquant de nombreuses autres pathologies inflammatoires (cysticercoses et abcès a pyogènes) ou néoplasiques (métastases, gliomes ou lymphomes) [9–12].

**Figure 4:**
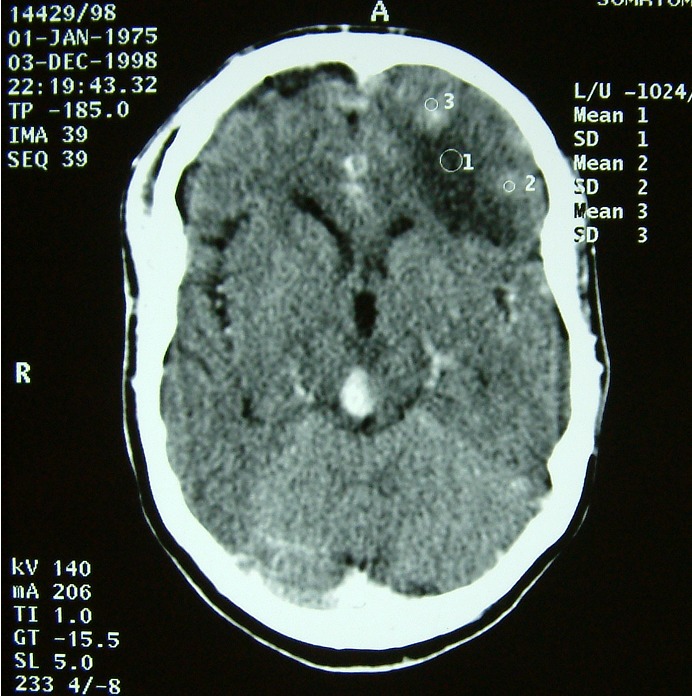
TDM cérébrale avant (à droite) puis après injection de produit de contraste (à gauche) montrant des tuberculomes multiples frontales et au niveau de la région pinéale sous forme de lésion spontanément hyperdense au sein d'une hypodensité périlésionelle, et qui se rehausse de façon hétérogène après injection de contraste

**Figure 5:**
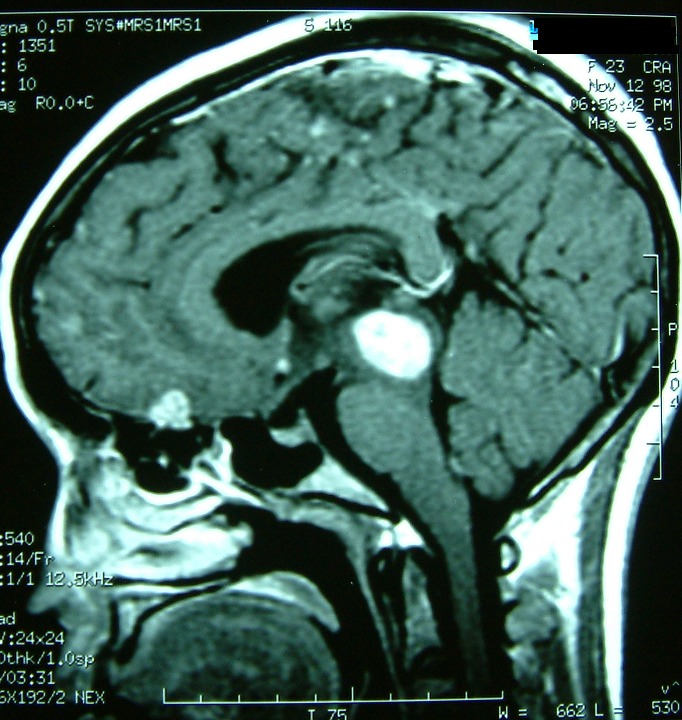
IRM cérébrale en séquences pondérée T1 après injection de gadolinium, et en coupes axiales (à droite) et en coupes sagittales (à gauche); montrant les tuberculomes sous forme d'hypersignaux en frontale gauche, région pinéale et cérébelleux

**Figure 6:**
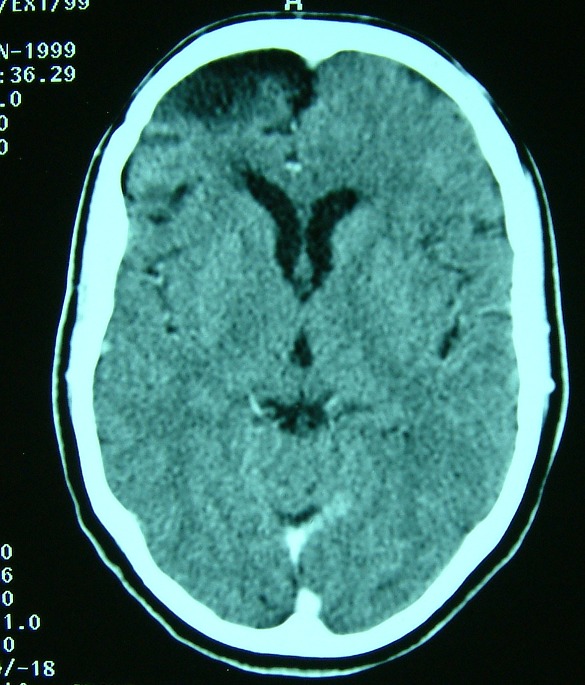
TDM cérébrale après injection de contraste montrant le nettoyage radiologique après abord chirurgicale de la lésion frontale et au terme du traitement antibacillaire. A noter la persistance de la cavité pore-encéphalique

En IRM séquence pondérée T1, le tuberculome donne un signal iso-intense à hypo-intense par rapport au parenchyme cérébral, et en séquence pondérée T2, un signal hypo-intense tandis qu'il existe à l'intérieur de la lésion plusieurs hypersignaux punctiformes [13]. Le tuberculome est entouré d'une zone irrégulière d'hypersignal en T2 correspondant à un oedème. Après injection de gadolinium, on observe une prise de contraste intense périphérique et circulaire. Le tuberculome intracrânien est le plus souvent unique; les formes multiples restent rares [1], dans notre série ces tuberculomes multiples représentent 6,4% des cas (Figure 4, Figure 5, Figure 6). Avant 1993 notre attitude thérapeutique consistait soit en un abord direct de la lésion dans 70% des cas, soit un traitement antituberculeux en première intention sans confirmation histologique (30%). Cette attitude était corrélée à une mortalité et morbidité non négligeables respectivement 3% et 10%. Après introduction de la stéréotaxie dans notre protocole de prise en charge; le taux d'abords direct a chuté a 39%, avec 48% de biopsies stéréotaxiques et seulement 13% des patients traités par antibacillaire sans preuve histologique. Ceci a été corrélé a une réduction statistiquement significative de mortalité à 1,4% (p = 0,0027) et de morbidité à 2% (p = 0,0003) (Tableau 2). La rentabilité diagnostic de la biopsie stéréotaxique est variable en fonction des séries de la littérature allant de 28% dans la série de Rajeshkhar [14] a 85% de Aaron Mohanty [15]. Dans notre série le taux de confirmation histologique par biopsie stéréotaxique est de 89%; ceci est peut être liée a la pratique de plusieurs prélèvements biopsiques étagés allant du centre a la périphérie de la lésion.

La biopsie cérébrale en condition stéréotaxique est réputée être une procédure peu invasive avec un taux de complications variant de 0,6 à 6,3% en fonction des séries [16–18]. Dans notre expérience nous n'avons eu aucun incident lié à cette procédure. Tous nos malades ont reçu le même protocole de chimiothérapie antibacillaire comportant 4 antibacillaires au doses suivantes: Isoniazide 5 mg /Kg/jour, Rifampicine 10mg/Kg/jour, Pyrazinamide 25 mg/Kg/jour et la Streptomycine 15mg/Kg/jour. La durée de traitement est en général de 9 à 12 mois selon l’évolution des lésions. Pendant les 2 premiers mois on associe les quatre antibacillaires puis 2 (Isoniazide et Rifampicine) pendant 7 à 10 mois. Des durées plus prolongées allant jusqúà 18 mois sont parfois nécessaires. Le traitement anticomitial est administré systématiquement et pour une durée minimale de deux ans. Etant donné le mécanisme physiopathologique du tuberculome et l’œdème associé, les corticoïdes sont justifiés dès la mise en route du traitement antibacillaire, ceci à la doses de 1 à 2 mg /Kg /jour.

**Figure 7:**
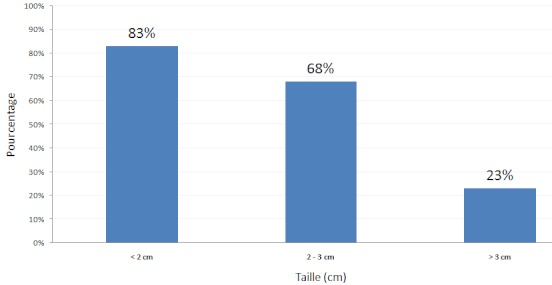
Taux de tuberculomes ayant diminués leurs tailles de moitié a 6 mois de traitement antibacillaire en fonction de leur taille initiale

L’évolution sous traitement se fait sous surveillance des paramètres cliniques et biologiques mais surtout sur les scanners de control. En général on constate une réduction de l’œdème péri-lésionnel et la taille des lésions dès la huitième semaine de traitement. Par la suite l'involution progressive du tuberculome se fait sur plusieurs mois. La vitesse d'involution des tuberculomes semble liée a leurs taille; ainsi dans notre série; le taux de tuberculomes ayant diminués leurs diamètres de plus de 50% au bout de 6 mois de traitement est de 83% dans le groupe de moins de 2 cm, 68% dans le groupe de 2-3 cm et seulement 23% pour les tuberculomes de plus de 3 cm (Figure 7). Pour Awada [3] la vitesse de régression des tuberculomes sous traitement antibacillaires est lente durant le premier mois du traitement, elle s'accélère entre le 2^ème^ et le 5^ème^ mois puis se ralentis après le 6^ème^ mois, puis disparition de toute image de tuberculome au 11^ème^ mois. Notre protocole de prise en charge actuel est superposable a celui d’ Aaron Mohanty [15], il est basé sur la nécessité d'obtenir une preuve histologique. Le traitement antibacillaire présomptif est réservé aux cas ou il existe un contexte tuberculeux manifeste avec BK positifs et contre indication à la chirurgie. L'abord chirurgical direct ne se justifie qu'en cas d'hypertension intracrânienne menaçante, altération de l'acuité visuelle, hydrocéphalie sur tuberculome de la fosse postérieure et l'augmentation paradoxale de la taille sous traitement médical [19, 20]. Dans les autres cas la suspicion de tuberculome représente une indication de choix pour la biopsie stéréotaxique [21, 22]. A la lumière de nos résultats nous préconisons l'arbre décisionnel pratiqué dans notre service (Voir arbre décisionnel). On a eu 5 cas de récidives (4%) dont 2 survenus plusieurs mois après arrêt du traitement et trois liées a un arrêt intempestif des antibacillaires avant le 6^ème^ mois de traitement. dans tous ces cas l’évolution était favorable après reprise du traitement antibacillaire avec une durée plus prolongée.

## Conclusion

Le tuberculome intracrânien est une lésion grave dont l'incidence reste élevée dans les pays d'endémie tuberculeuse. Le diagnostic positif est basé sur un faisceau d'arguments de présomption clinico-biologiques et radiologiques, la confirmation reste histologique. Aucune image radiologique n'est spécifique du tuberculome. Actuellement avec le développement de la stéréotaxie l'abord chirurgical direct des tuberculomes ne se justifie qu'en cas de lésions avec hypertension intracrânienne menaçante. La biopsie stéréotaxique constitue la procédure de choix en cas de suspicion de tuberculome avec une forte rentabilité diagnostic et une faible morbidité. Le traitement antibacillaire entrepris précocement permet une guérison avec peu de séquelles.

## Annexe

**Annexe 1:** Algorithme montrant notre protocole actuel de prise en charge des tuberculomes intracrâniens.
